# *Fastosh*: a software for the treatment of XAFS datasets of environmental relevance or acquired in *operando* conditions

**DOI:** 10.1107/S1600577525003923

**Published:** 2025-06-23

**Authors:** Gautier Landrot, Emiliano Fonda

**Affiliations:** a Synchrotron SOLEIL L’Orme des Merisiers, Départementale 128 91190Saint-Aubin France; University of Essex, United Kingdom

**Keywords:** general-purpose XAFS data treatment software, study of mixtures via chemometry, kinetics dataset tools, quick-EXAFS modelling, HDF file processing

## Abstract

*Fastosh* is a freeware dedicated to the treatment of X-ray absorption fine-structure (XAFS) spectroscopy data. The program features functions that are particularly suitable for geochemical and environmental applications and treatment of XAFS datasets acquired in *operando* conditions. It can be employed to identify the nature of chemical species present as principal components in a sample mixture, which is a case commonly encountered when studying a set of natural samples or a chemical reaction followed *in situ* at the beamline, where multiple species may coexist, including intermediary species. Additionally, the software features unique functionalities that notably allow to filter in two dimensions and plot in three dimensions time-dependent datasets; rapidly deglitch chi spectra; automatically view in 2D or 3D and merge XAFS data acquired at the beamline; and visualize an EXAFS wavelet transform map as well as interpret it and its associated chi and Fourier transform spectra using a quick modelling approach where theoretical single scattering paths are employed. All these functions can be applied to XAFS data generated in ASCII format at any XAFS beamlines. Lastly, the program specifically allows users of SAMBA beamline, Synchrotron SOLEIL, to easily access all contextual data associated with an XAFS acquisition or process the raw data collected by the multi-pixel fluorescence detector of the beamline, which are saved in an HDF file. The structure of this file, and all related functions in *Fastosh* to exploit it, could be readily adapted to comply with a universal HDF format, which hopefully will be defined and adopted in the future by multiple XAFS beamlines worldwide.

## Abstract 2

The main functionalities of *Fastosh*, especially those that are unique to the program, are presented. This notably includes various tools based on chemometry (*e.g.* MCR-ALS) and some functions to interprete the Fourier or wavelet transform using a quick-EXAFS modelling approach.

## 1. Introduction

*Fastosh* was conceived at SAMBA, a beamline dedicated to bulk XAFS in the hard X-ray domain (*i.e.* 5 to 40 keV) at Synchrotron SOLEIL, France (Briois *et al.*, 2011 ▸). The name of the program is pronounced [*fast-osh*] as the French word ‘fastoche’, which familiarly means in French language ‘easy’. The first version of the program was written from 2016 to 2018. The creation of the software was originally motivated to provide solutions to various limitations encountered, prior to the existence of this program, as a result of SAMBA beamline evolution, or in various XAFS data treatment steps, including limitations in the codes already available or lack of algorithm accessibilities. For example, when a continuous acquisition mode was introduced at SAMBA in 2016, as an alternative method to the older step-by-step acquisition mode of the beamline, there was a need at SAMBA for a multi-purpose XAFS data treatment software, with a friendly graphical user interface, to rapidly load an XAFS spectrum that may contain several thousand datapoints, with no limit on the number of spectra to import, and with adapted program functionalities to process, in multiple ways, large datasets.

Identifying the nature of the main chemical species relative to a studied element and present in a given sample mixture is an issue that can be encountered in many fields of science, including those where the XAFS technique is employed. This is particularly the case in environmental/geochemical studies, since natural samples do not usually constitute a single pure chemical species, and when a chemical reaction is followed *in situ* at the beamline, where multiple species may co-exist at the same time including intermediate species. To address this challenge, two approaches applicable to spectroscopic data have been proposed, the Target Transformation (TT) and Multivariate Curve Resolution–Alternated Least Square (MCR-ALS) methods, whose principle were described by Malinowski (1978 ▸) and Jaumot & Tauler (2010 ▸), respectively. To apply any of these methods on a given dataset constituting multiple sample spectra, one must fundamentally assume that the same chemical species entirely make up the dataset but in specific proportions in each sample. To our knowledge, the tools available to perform TT to XAFS spectra prior to the creation of *Fastosh* only allowed a SPOIL value to be determined, whose calculation is detailed by Malinowski (1978 ▸) and summarized in Fig. S1 of the supporting information, one reference compound at a time, instead of calculating the SPOIL values on an entire standard library. This may not be convenient given that the standard library may feature more than a dozen references, such as the one employed in the study by Doelsch *et al.* (2024 ▸). Additionally, to our knowledge, no general-purpose XAFS data treatment software that were available prior to the creation of *Fastosh* featured MCR-ALS capabilities. While the toolbox GUI 2.0 of Jaumot *et al.* (2015 ▸) represents one of the most advanced algorithms to perform MCR-ALS on spectroscopic data, it must be employed inside the Matlab software under an active MathWorks licence. Therefore, providing solutions to these chemometry limitations represented one of the original goals that motivated the creation of the program.

Since the birth of *Fastosh*, it has been employed in various fields of science, including environmental (Doelsch *et al.*, 2024 ▸; Pons *et al.*, 2021 ▸; Tella *et al.*, 2023 ▸; Blommaert *et al.*, 2024 ▸; Garnier *et al.*, 2024 ▸; Blommaert *et al.*, 2022 ▸; Sricharoenvech *et al.*, 2024 ▸), food (Schiell *et al.*, 2025 ▸; Rivard *et al.*, 2024 ▸), catalysis (Dolcet *et al.*, 2021 ▸; De *et al.*, 2021 ▸; Dokania *et al.*, 2021 ▸), electrochemistry (Saïbi *et al.*, 2023 ▸; Saïbi *et al.*, 2024 ▸) and material science (Vanni *et al.*, 2023 ▸). Also, multiple software functionalities have been progressively added or modified, including those meant to visualize an EXAFS wavelet transform map as well as rapidly interpret it and its associated chi and Fourier transform spectra. The goal of this communication is to present these functions, especially those that are unique to the program.

## 2. Overall presentation

*Fastosh* is coded in Matlab language (MathWorks). The latest available version (*Fastosh* v1.0.8) was written with Matlab 9.14 (R2023a). It is compiled for Windows, MacOS and Linux operating systems. On MacOS systems it has been successfully tested on Catalina 10.15.7 (Intel Chip, 2018) and Sonoma 14.5 (silicone chip, 2024). The current version of the software, its user manual and source code are available for free download on the SAMBA beamline website (https://www.synchrotron-soleil.fr/en/beamlines/samba). The program is distributed under a GNU GPL-3.0-or-later licence.

The installer of the program comes as a single file, regardless of the type of operating system. It features inside it all components to run every functionality of *Fastosh*. Indeed, the installer includes, in addition to the functions that are specific to *Fastosh*, a customized version of the MCR-ALS toolbox 2.0 of Jaumot *et al.* (2015 ▸), a static version of *FEFF8L* compiled in Fortran language (Ankudinov *et al.*, 1998 ▸), as well as some functions of *Larch* (Newville, 2013 ▸) for the pre-data treatment steps, *e.g.* normalization, background subtraction, visualization of Fourier and reverse Fourier transform. These *Larch* functions were entirely translated to Matlab language to optimize the speed and installer size of the program, so that the software could run exclusively under a Matlab environment. Lastly, the installer features a Matlab Runtime, so that *Fastosh* can be employed as a standalone software using all the required Matlab functions without the prerequisite of owning a MathWorks licence.

## 3. Description of functionalities

The main functionalities of the program are described here. The first section focuses on the functions that are employed during the pre-data treatment, between data importation and exploitation (Section 3.1). The second part describes the main program functions that can be employed once the pre-data treatment step is completed in order to gain quantitative and/or qualitative information from the XAFS data (Section 3.2). Most functionalities of the program, including all those detailed in Sections 3.1 and 3.2, are compatible with data saved in ASCII format, which may optionally feature an informative header. The data imported to *Fastosh* can be raw mu, normalized mu or chi spectra. For MCR-ALS processing, non-XAFS data can be also imported into *Fastosh*, which also features specific pre-data treatment functions for such data type (Section 3.1.2). Lastly, a few functions of *Fastosh* are exclusively compatible with HDF files generated at SAMBA. Their nature and potential benefits are detailed in the last section (Section 3.3).

### 3.1. Pre-data treatment

#### 3.1.1. Basic functions

**Figure 1:**
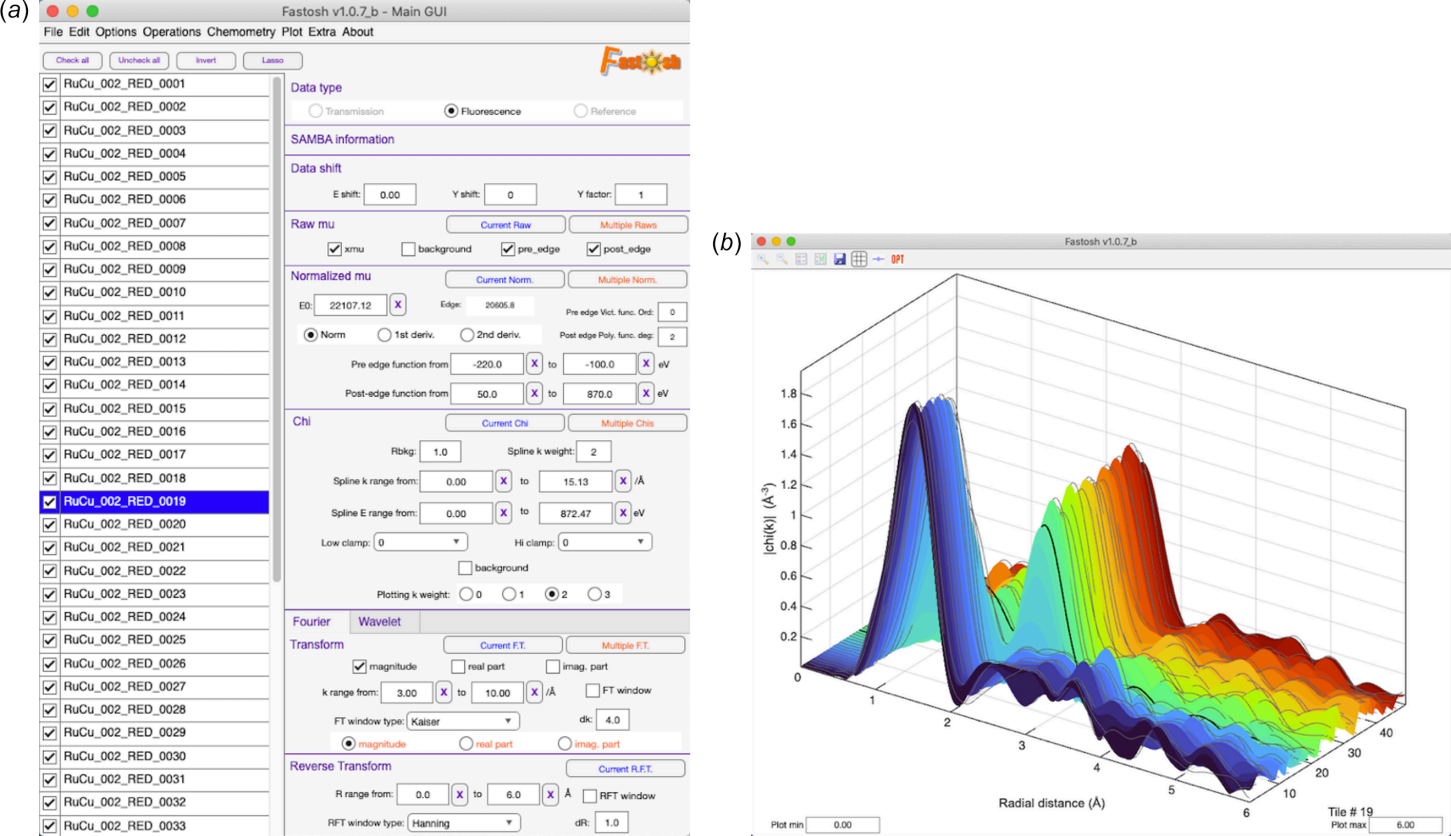
Main GUI (*a*) and associated plot window (*b*).

All basic pre-data treatment steps can be found in the main GUI of the software [Fig. 1 ▸(*a*)]. For datasets constituting short spectra that are difficult to normalize using the *Larch* normalization approach, alternative normalization methods are available: those featured in the program *PyMCA* (Solé *et al.*, 2007 ▸) and one method exclusive to *Fastosh* where the beginning of all spectra are set to 0 and a user-defined region, towards the end of all spectra, is vertically equalized to 1 using a least-squares fitting approach. The program proposes a number of functionalities that are particularly useful for plotting large and/or time-dependent datasets. For example, time-dependent datasets can be plotted in 3D. An option allows to highlight in the 3D plot the specific spectrum that is currently selected in the sample list featured in the main GUI [Fig. 1 ▸(*b*)]. Another option allows to plot, in 2D or 3D, the difference between the first spectrum and each remaining spectrum of a dataset, when the mu, chi or Fourier transform are displayed. This can be useful for revealing small spectral differences occurring in the dataset. Another useful functionality called ‘Lasso’ enables many samples that are successively displayed in the sample list of the main GUI to be automatically checked, to avoid manually checking each of them. When employed, it prompts to manually check only two samples available anywhere in the sample list. Once these two samples are selected, the function will automatically check all samples that are listed between them.

The program features all expected basic functions for pre-data treatment of XAFS spectra, including a dedicated module for calibration and alignment, data trunction, data interpolation and data deglitching using a manual approach. It also contains functions for XAFS pre-data treatment that are less commonly found in XAFS data treatment software, which are individually presented in the next section.

#### 3.1.2. Specific functions

*Live data viewer and merger*. This module enables to visualize, in 2D or 3D, the merge spectrum (raw mu, normalized, chi, or Fourier transform spectrum) or all associated individual iteration spectra, whose corresponding data files are located in a specific data folder. This tool is automatically and periodically refreshed (Fig. S2). It can also show the progressive improvement of the estimated random noise of the EXAFS, via the well documented *Larch* method (Newville, 2013 ▸), and signal-to-noise ratio of the XANES, by quantifying the difference between the experimental data at the beginning of the spectrum where the signal is flat and its noise-free equivalent that is obtained by fitting the experimental spectrum using a polynomial function of degree two, for a set of spectra corresponding to a specific sample [Fig. S2(*c*)]. Therefore, this module can be employed directly at the beamline as a fully automatic tool to display all acquired XAFS spectra, or their corresponding average spectrum, corresponding to the sample being analysed. It can thus help determine when enough data have been collected for the sample being analysed, which can be particularly useful for diluted samples requiring long acquisition times [Fig. S2(*b*)]. It can be also utilized to automatically follow an *operando* reaction taking place in real time at the beamline, where XAFS spectra shapes are expected to evolve with time [Fig. S2(*a*)].

*Chunk merging*. When a time-dependent dataset features many iterations, it may be reduced to a smaller dataset. Each element of the new dataset corresponds to the merging of a specific number of iterations featured in the original dataset, which is a user-defined number. For example, a dataset constituting 616 XAFS spectra collected in *operando* conditions at a quick-XAFS beamline can be reduced to 56 spectra, by merging every 11 spectra consecutively acquired at the beamline (Fig. S3). Therefore, this module may be useful to increase the signal-to-noise ratio of the original data, which, in turn, may notably improve the speed of all subsequent analyses where the dataset is exploited.

**Figure 2:**
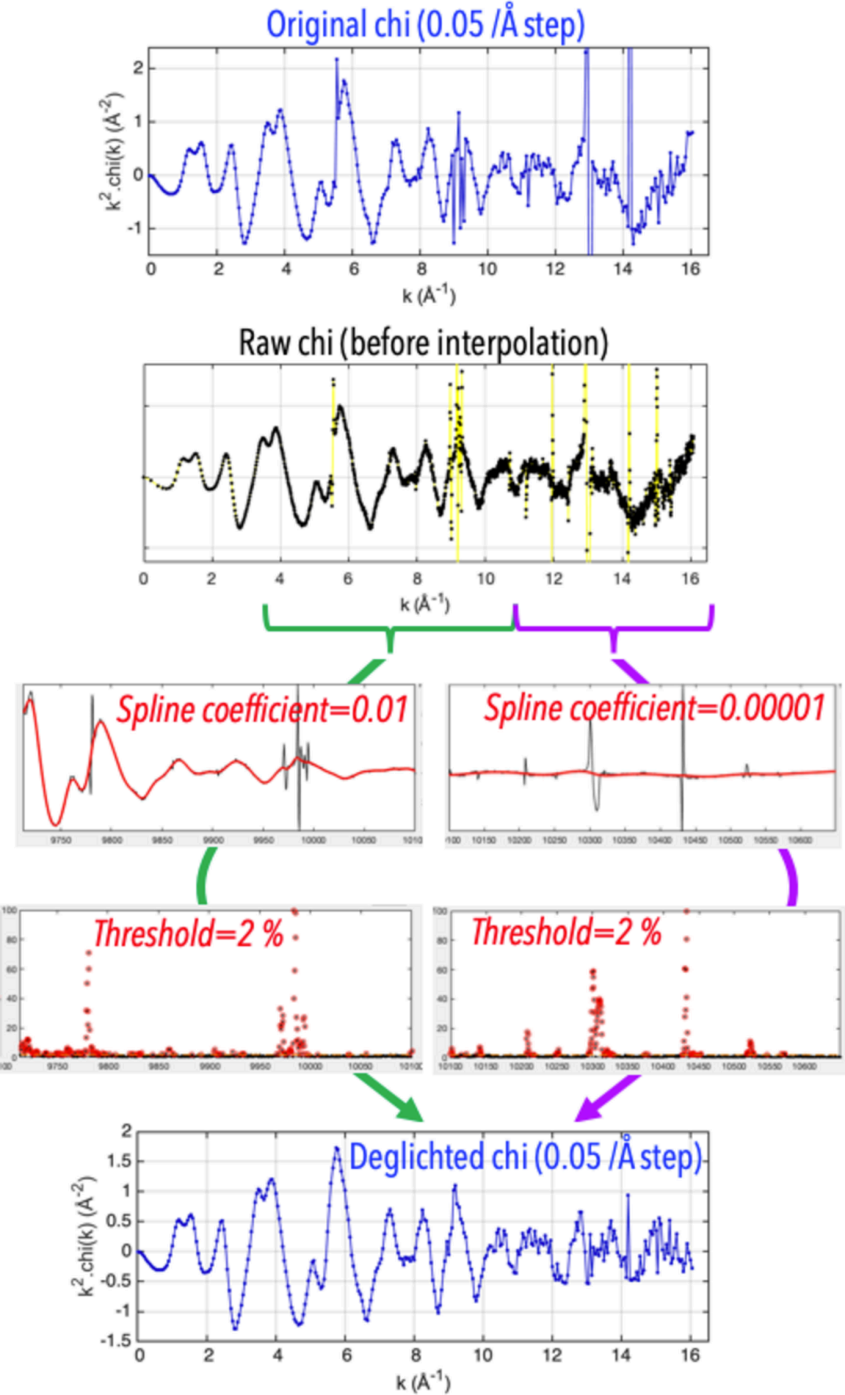
Autodeglitching example.

*EXAFS autodeglitching*. A function called ‘autodeglitching’ proposes a deglitching method that is more automatized than the manual deglitching approach. With this method, the raw chi spectrum featuring all data points (*i.e.* before chi is interpolated into a constant 0.05 Å^−1^ step grid in *k* space) is fitted at a specific region with a smoothing spline function. At each *k* value in this region, the modulus of the experimental value is subtracted by the value of the fitted function. If the resulting value is above a user-defined threshold value, which by default is equal to four times the standard deviation of all data points, the experimental data point is considered as a glitch and removed from the raw data (Fig. 2 ▸). Spectra acquired with a continuous acquisition or QXAFS mode are particularly well suited for this type of deglitching approach as they typically featured many datapoints, up to several thousands. One can thus apply to such spectra a very low threshold value to remove many problematic datapoints, while still keeping enough data to obtain satisfactory deglitching results as demonstrated in Fig. 2 ▸.

*2D filtering*. A function allows to simultaneously smooth in two dimensions, *i.e.* following both the energy and tile directions [Fig. S4(*a*)], the normalized mu or chi spectra belonging to a given time-dependent dataset, using a 2D Savitzky-Golay filter. The nature of the filter is well adapted to reduce the noise of the signal while not affecting the structural oscillations. Therefore, this filter is mainly employed for cosmetic purposes, to improve the appearance of a time-dependent dataset plotted in 3D, such as the one shown in Fig. 1 ▸(*b*) and Fig. S4(*b*), before and after application of the 2D filter, respectively.

**Figure 3:**
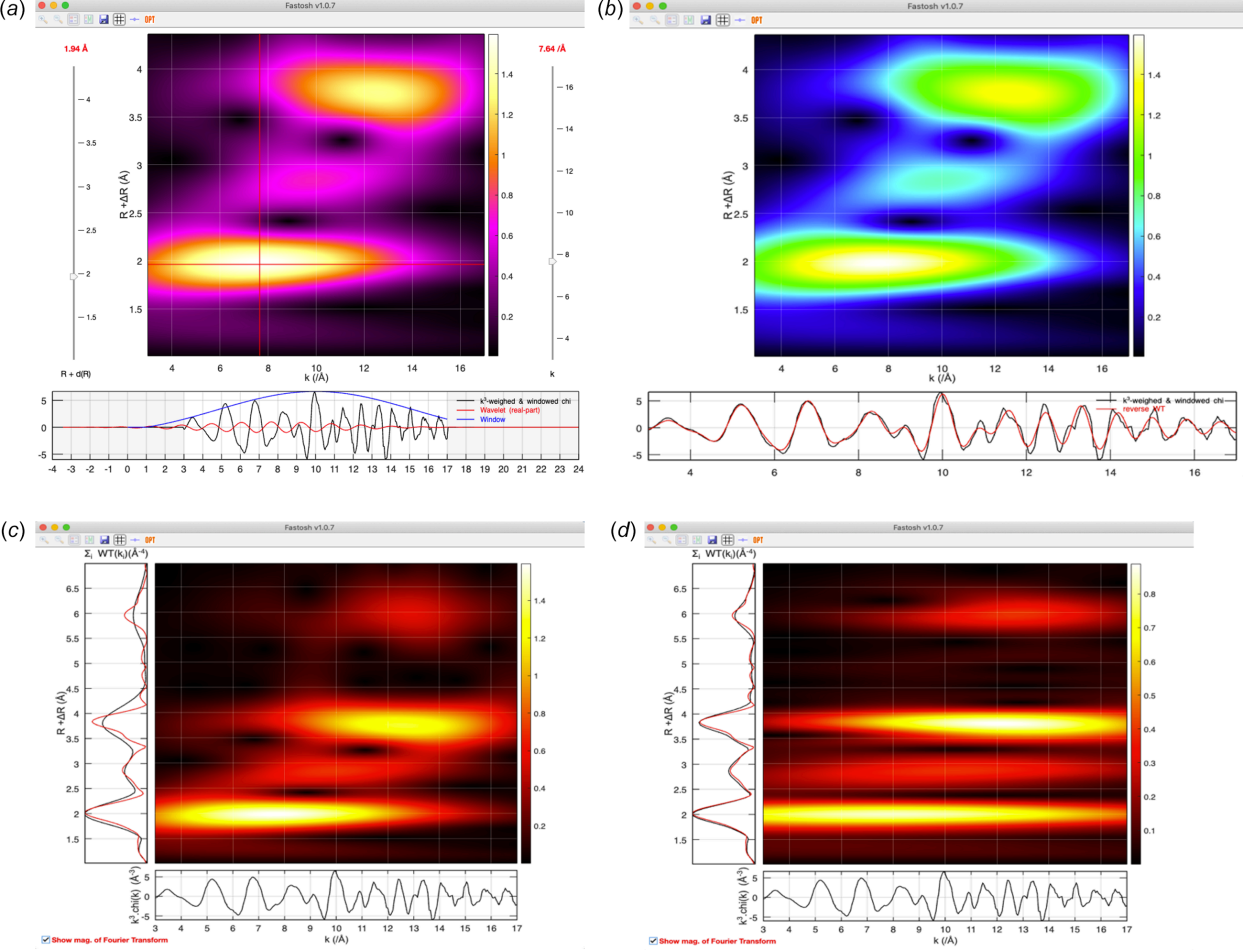
Wavelet transform plotting options: interactive window featuring the wavelet that is employed to process the chi for a given set of *R* and *k* values (*a*), window featuring the reverse WT spectrum (*b*), and window allowing the resolution in *R* space between the FT and WT to be compared, where the Cauchy *n* parameter was set to its default value (*c*) or fitted to equalize the WT and FT resolution in *R* space (*d*).

*Wavelet transform (WT) of the EXAFS*. The EXAFS data can be processed via a WT approach, as an alternative method to the Fourier transform (FT) approach. The Cauchy and Morlet wavelets are the two types of wavelet that have been mainly employed to process EXAFS spectra. Both were originally introduced many years ago, by Munoz *et al.* (2005 ▸) and Funke *et al.* (2005 ▸), respectively. However, tools available nowadays to perform such operations are still scarce. The Cauchy and Morlet wavelets are available in *Fastosh*, which features multiple plotting functions to display the WT results. For example, one interactive functionality can be employed as an educational tool to visually understand the relationship between the specific *k* and *R* values at each pixel of the WT map and the wavelet’s frequency and centre position in *k* space, as well as the effects of the wavelet parameter (Cauchy’s *n*, or Morlet’s eta/sigma) on the shape of the wavelet used to process the EXAFS [Fig. 3 ▸(*a*)]. The user-defined values corresponding to these wavelet parameters, *k* range and *R* range of the WT, and type of apodization window that is multiplied by the chi to obtain the EXAFS employed in the WT, are all available in a block dedicated to WT inside the main GUI. Another plotting option allows the reverse WT spectrum to be displayed along with the WT map [Fig. 3 ▸(*b*)]. Lastly, the EXAFS FT spectrum can be plotted along with the chi spectrum containing the EXAFS and the EXAFS WT map, similarly to the original WT toolbox by Munoz *et al.* (2005 ▸). The plot window also features, in a vertical plot where the FT spectrum is displayed in red, a spectrum displayed in black, where each datapoint corresponds to the sum of each pixel row of the WT map [Figs. 3 ▸(*c*) and 3(*d*)]. This enables the resolution in *R* space between the WT and FT to be compared. For example, when a Cauchy WT was applied to the EXAFS of thorite collected at the thorium *L*3 edge, using a Cauchy *n* parameter equal to 200, which is the default value for this wavelet type, the resolution in *R* space of the WT was poorer compared with that of the FT [Fig. 3 ▸(*c*)]. A fitting tool available in *Fastosh* enables to minimize, using a least-squares fitting procedure where the Cauchy *n* WT parameter is floated, the difference between the FT spectrum and that where each datapoint corresponds to the sum of each pixel row of the WT map. For example, it was found, using this tool, that the resolution in *R* space between the FT and WT of thorite could be equalized when the Cauchy *n* parameter was equal to 1046 [Fig. 3 ▸(*d*)]. Although such a value significantly decreased the resolution in *k* space of the WT map, which was inevitable as the resolutions in *R* and *k* spaces of a WT map are inversely proportional (Funke *et al.*, 2005 ▸), the different intensity maximums occurring at specific *R* distances could still be observed at specific values in *k* space in the WT map. For instance, the intensity maximums occurring vertically in the WT map at 2 and 3.8 in *R* space (uncorrected for phase shift) were still observed horizontally in the WT map around 8 and 12 Å^−1^, respectively [Fig. 3 ▸(*d*)].

*Pre-data treatment of non-XAFS data*. A functionality that multiplies the potential usefulness of the program enables to import non-XAFS data (*e.g.* Raman, infrared, X-ray diffraction) to *Fastosh*, so that it can be eventually processed by MCR-ALS. This includes data acquired at a synchrotron facility or benchtop laboratory device, and optionally in two dimensions using a mapping mode [Fig. S5(*b*)]. Similarly to an XAFS spectrum, the variables and observations of the non-XAFS spectrum to import to *Fastosh* must be found in specific data columns in the ASCII file. Pre-data treatment functions for such data type include data truncation and baseline substraction using the algorithm developed by Mazet *et al.* (2005 ▸), which was designed to be applied to various types of spectroscopic signal [Fig. S5(*a*)].

### 3.2. Quantitative and qualitative analyses

Once all pre-data treatment steps have been completed, the functions described in each section below can be applied to XAFS spectra to obtain quantitative and/or qualitative information on the samples.

#### 3.2.1. Plot of *R*-factor or residual values following multiple linear combination fitting

*Fastosh* proposes two main approaches to perform linear combination fitting (LCF) (Fig. S6). The first one consists of fitting a single sample spectrum by LCF using a set of references, or optionally using multiple combinations of references. The latter option can be employed when the sample to fit by LCF is part of a dataset corresponding to a sample mixture where the number of principal components is known, but their specific chemical nature is unknown. For example, if the sample mixture allegedly constitutes four principal components, and there are eight references available to perform LCF, the program will perform 70 LCF operations on a single sample belonging to the sample mixture, using the 70 possible unique combinations of four references [Fig. S6(*a*)].

The second LCF approach proposed in *Fastosh* is the possibility to fit by LCF multiple samples, using a single set of references [Fig. S6(*b*)]. This method can be applied when the samples are part of a dataset where both the number of principal components and their chemical nature are known, *i.e.* when a TT or MCR-ALS approach has been employed prior to performing LCF (Section 3.2.2). Additionally, this second LCF approach can be applied to a kinetics dataset to provide information on the number of main chemical species that are coexisting at a given time in the system. For example, it was employed to process a dataset constituting 28 time-dependent XAFS spectra, collected during an *operando* experiment carried out at the beamline at the Cu *K*-edge, where Cu was reduced.

**Figure 4:**
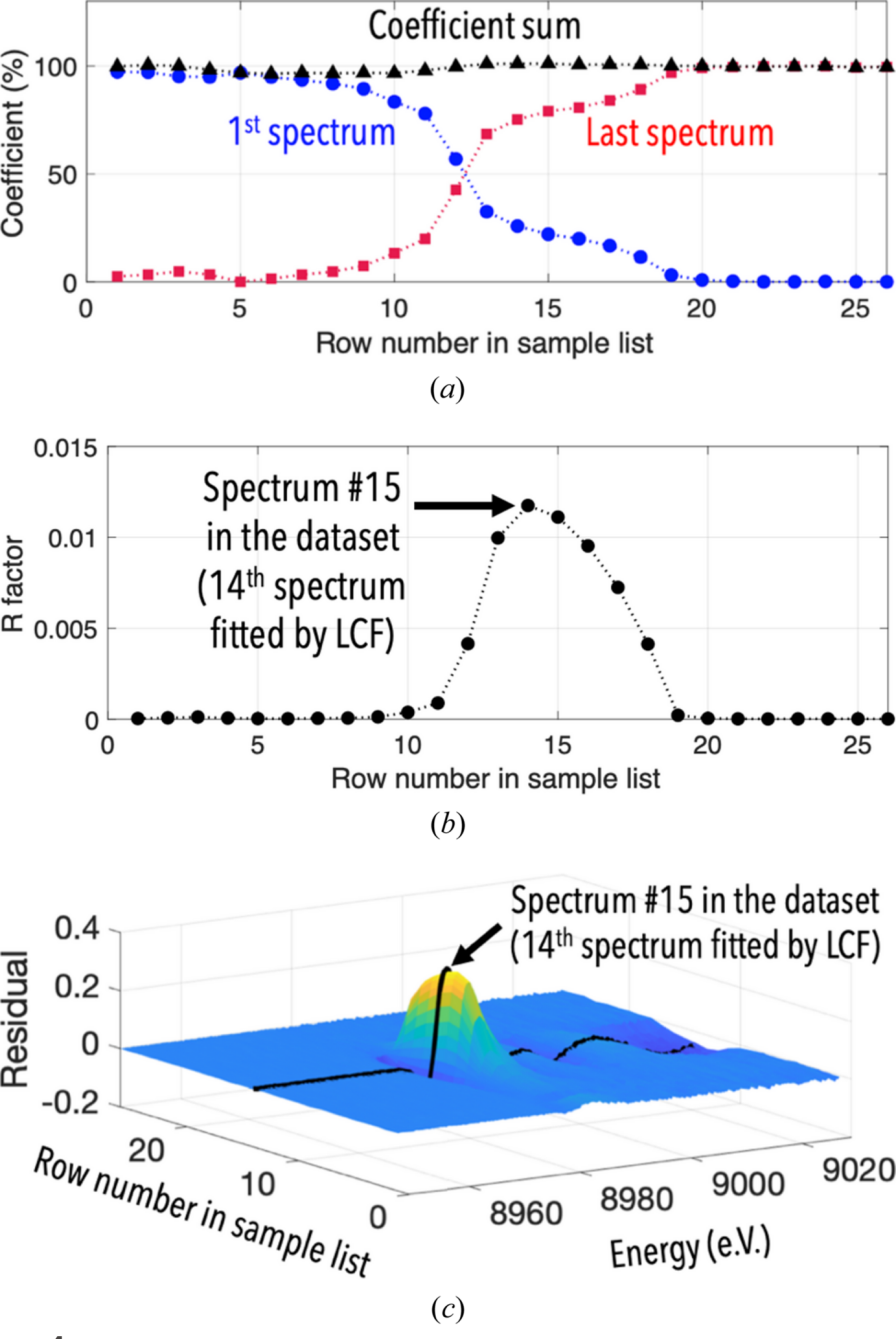
LCF coefficients (*a*), *R*-factor (*b*) and residual (*c*) obtained after performing, on a dataset consisting of 28 time-dependent XAFS spectra, LCF to all spectra from the 2nd to the 27th using as references the 1st and 28th spectra.

**Figure 5:**
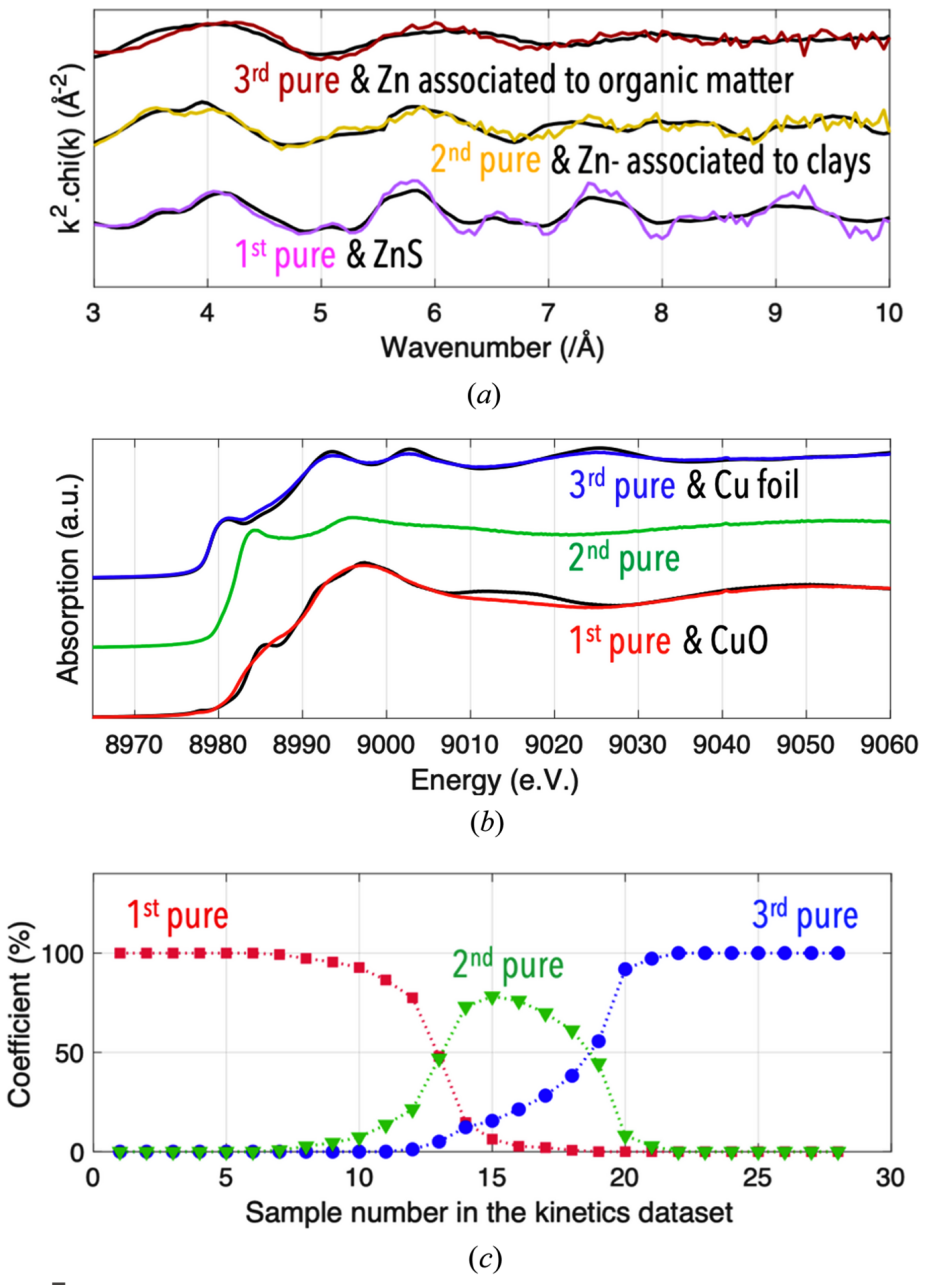
Pure chi spectra obtained by treating by MCR-ALS a natural dataset constituting EXAFS spectra corresponding to zinc-polluted sediments (*a*); normalized mu spectra (*b*) and relative concentration profiles (*c*) determined by MCR-ALS applied to the same kinetics dataset as the one employed previously in the multiple LCF example (Fig. 4 ▸). The pure spectra associated with the former and latter datasets are displayed along with specific zinc and copper reference compounds, respectively, which were identified using the post MCR-ALS module of *Fastosh*.

Multiple LCF operations were performed on all spectra of the dataset, from the 2nd to the 27th iteration, using two reference spectra. The latter were the 1st and 28th (*i.e.* first and last) iterations of the dataset, assuming that they corresponded to a reactant and product of the chemical reaction, respectively. While the sum of the coefficients obtained for each of these LCF operations was systematically equal to around 100% [Fig. 4 ▸(*a*)], the *R*-factors and residual values corresponding to the LCF fitting of spectra 12–18 were much higher than those obtained with the remaining spectra of the dataset [Figs. 4 ▸(*b*) and 4(*c*)]. The highest *R*-factor and residual values were obtained at the 14th LCF operation, corresponding to the 15th sample spectrum of the kinetics dataset. This suggested that a third species temporarily existed during the course of the reaction and reached a maximum level when the 15th sample spectrum was collected. This was subsequently confirmed when the MCR-ALS method was applied to this dataset, as demonstrated later on [Fig. 5 ▸(*c*)]. Therefore, this LCF approach may be employed prior to performing MCR-ALS as an alternative method to PCA to determine the number of principal components present in the system, which must be provided as an input parameter at the onset of the MCR-ALS procedure. In contrast to the possibility of reporting on a single plot all the coefficients obtained from an LCF of multiple samples, the ability to display on a single plot all the *R*-factor or residual values obtained from LCF of multiple samples is less commonly found in XAFS data treatment software, despite the potential usefulness of such an approach as illustrated in Fig. 4 ▸.

#### 3.2.2. Chemometric methods

The *Fastosh* module dedicated to PCA and TT allows to calculate all at once the SPOIL value associated with all standards that are part of a personal reference library, instead of processing them one at a time. For example, it was employed to calculate all at once the SPOIL values of 41 copper (Cu) references in an effort to elucidate the main chemical forms of Cu present in organic wastes affecting agricultural soils (Doelsch *et al.*, 2024 ▸).

Additionnal features were added to the toolbox of Jaumot *et al.* (2015 ▸) to optimize and facilitate the MCR-ALS processing. This includes a function that automatically sets all the ALS refinement constraints that are typically applied to mu or chi spectra [Fig. S7(*a*)] and added GUIs for interactively constraining the spectrum of each pure species of the dataset [Fig. S7(*b*)], or coefficient of each pure species in a sample [Fig. S7(*c*)]. Another function added to the original toolbox allows evolving factor analysis (Maeder & Zilian, 1988 ▸) to be performed on an ‘augmented’ dataset, *i.e.* a dataset that features more than one time-dependent dataset, which may share the same principal components [Fig. S7(*d*)]. Lastly, a function that was not available in the original toolbox allows a composite map of all pure species to be created, for datasets acquired in 2D. Once the pure spectra corresponding to all principal components have been extracted by MCR-ALS, a post-MCR-ALS functionality featured in *Fastosh* can help identify these spectra by quantitatively comparing them, by means of *R*-factor calculation, with those belonging to a personal standard library (Fig. S8).

Results from MCR-ALS may enable the nature of the main chemical forms present in a sample mixture to be constrained more precisely, compared with those obtained with the TT approach. For example, results obtained from principal component analysis of EXAFS spectra indicated that there were three zinc (Zn) species mainly present in a set of Zn-polluted sediments collected in Sepetiba Bay, Brazil (Garnier *et al.*, 2024 ▸). The TT applied to this dataset could disqualify only three Zn references from a standard library featuring eleven Zn references, implying that all remaining standards could potentially correspond to one of the three principal components. In contrast, the nature of the three main Zn forms present in the sediments could be more accurately determined when the principal component spectra extracted by MCR-ALS were quantitatively compared with those corresponding to multiple Zn references, using the post MCR-ALS function of *Fastosh* [Fig. 5 ▸(*a*)].

The MCR-ALS approach is very well suited for the treatment of kinetics XAFS datasets, as demonstrated by Dolcet *et al.* (2021 ▸) or De *et al.* (2021 ▸). Indeed, it allows to obtain not only the spectra corresponding to the main species that coexist during a chemical reactions, including intermediate species [Fig. 5 ▸(*b*)], but also their relative levels present in the system when each spectrum was acquired [Fig. 5 ▸(*c*)]. For example, when the MCR-ALS method was applied to the kinetics dataset mentioned earlier corresponding to a chemical reaction where Cu was reduced in *operando* conditions at the beamline, the relative levels of the intermediate species determined by MCR-ALS [Fig. 5 ▸(*c*)] were, as expected, consistent with the *R*-factor trend obtained via the multiple LCF approach described earlier [Fig. 4 ▸(*b*)]. Additionally, results obtained via the post MCR-ALS tool suggested that the first and third pure species could correspond to a form similar to copper oxide (CuO) and metallic Cu, respectively [Fig. 5 ▸(*b*)]. However, this tool could not identify the chemical nature of the intermediate species of the reaction, since its corresponding spectrum could not match any available Cu standard spectra. As shown by Kim *et al.* (2003 ▸), it is not straightfoward to determine the chemical nature of an intermediate species that temporarily exist during a chemical reaction where Cu is reduced. Addressing this issue was possible in *Fastosh* using the MCR-ALS-extracted XAFS spectrum corresponding to the intermediate species, shown in Fig. 5 ▸(*b*), and the quick EXAFS modelling tool, as demonstrated in the next section.

#### 3.2.3. Quick modelling of the EXAFS

A tool enables the EXAFS of a sample to be modelled using theoretical chi spectra generated by *FEFF8L* and corresponding to single-scattering path models. Generating such models is thus very rapid and simple as it only requires the types of atom corresponding to the absorber and scatterer, shell distance and absorption edge to be provided. If multiple single-scattering models are created to reproduce a given experimental chi spectrum, each of them or their sum can be displayed in FT or WT space, and stacked on top of the experimental data. The fit parameters can be refined via a least-squares fitting procedure, where the fit minimization is achieved via FT or WT. Fitting can be done on a single chi sample spectrum, or multiple chi sample spectra, using a given theoretical model. In the latter case, once the fit is completed, all values obtained for a specific fit parameter can be plotted as a bar plot. Additionally, the values of all refined model parameters and goodness of fit can be displayed in a table, similarly to the way fit results are typically reported in research manuscripts. Lastly, the Hamilton test can be performed to determine whether two fit models, sharing the same fitting *k* and *R* ranges but with different number of fit variables, are significantly different from each other (Calvin, 2013 ▸).

**Figure 6:**
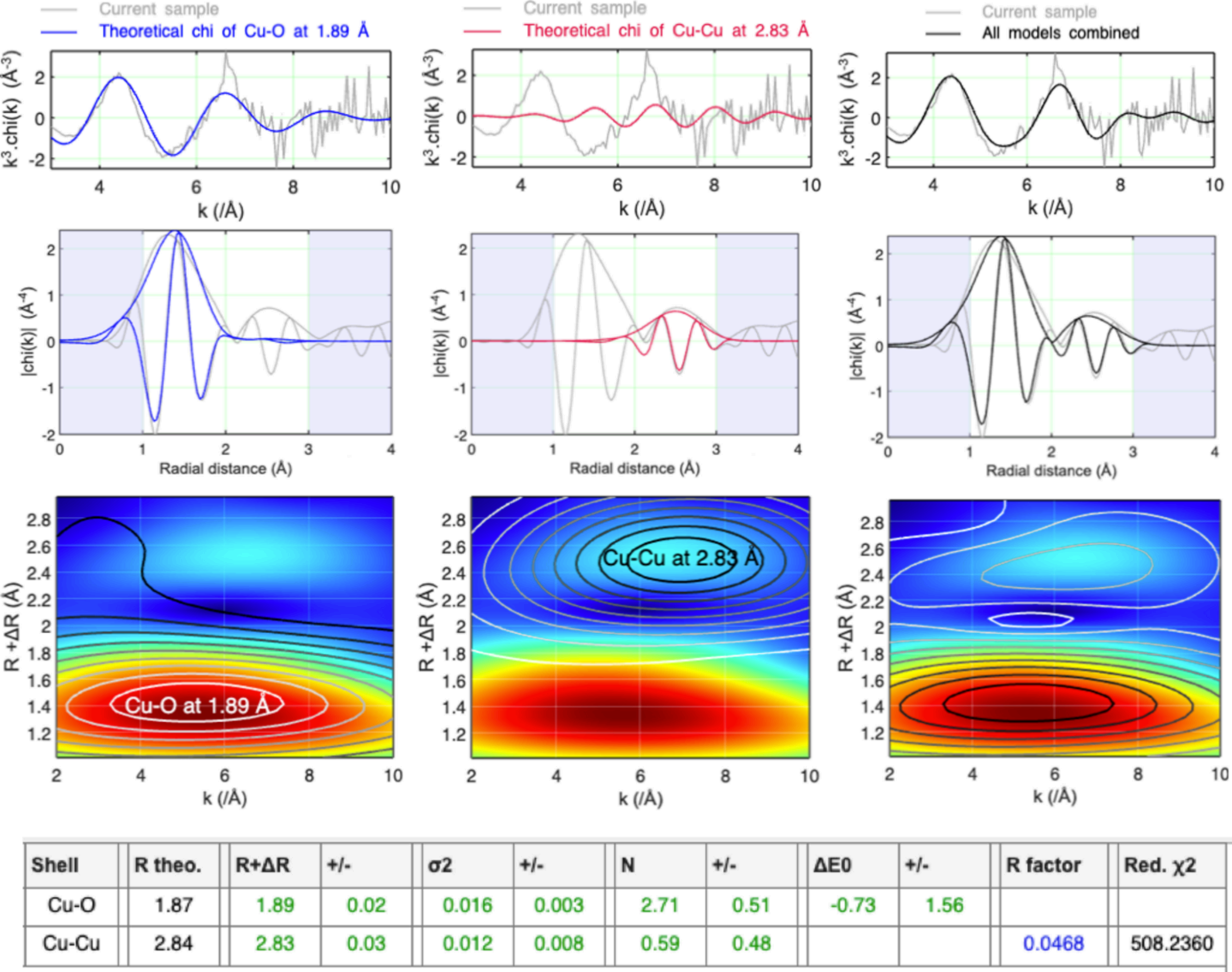
Fit results, corresponding to the intermediate species whose XAFS spectrum was extracted by MCR-ALS [Fig. 5 ▸(*b*)], obtained using the quick EXAFS modelling tool.

This tool is not meant to model an EXAFS spectrum where single and multiple scattering phenomena contribute to the signal. However, it may be utilized to conveniently interpret, for example, a region of a wavelet transform map where more than one scatterer may occur at a given distance around the absorber, or model an EXAFS spectrum where the use of only single scattering theoretical paths is permissible. This was possible, for instance, with the EXAFS spectrum corresponding to the intermediate species whose normalized mu was extracted by MCR-ALS from the kinetics dataset mentioned earlier [Fig. 5 ▸(*b*)]. This spectrum could be indeed theoretically reproduced using two single scattering models corresponding to Cu—O and Cu—Cu at 1.89 ± 0.02 and 2.83 ± 0.03 Å, respectively (Fig. 6 ▸). This was similar to the Cu—O and Cu—Cu shells at 1.94 and 2.82 Å, respectively, found in the mineral phase linarite [PbCu(SO_4_)(OH)_2_] (Schofield *et al.*, 2009 ▸). While the amplitude factor contributing to the EXAFS signal is by default defined in the *Fastosh* tool as the amplitude reduction factor (SO2) times the degeneracy N (*i.e.* ‘SO2 × N’), it can be optionally defined as ‘SO2’ or ‘N’. For example, it was defined as ‘N’ in the model mentioned above, and the value of SO2 was fixed to 1 (Fig. 6 ▸). The latter value was determined beforehand by modelling the first atomic shell around Cu in the structure of copper oxide (Cu_2_O), using the EXAFS corresponding to this chemical compound and a Cu—O single scattering model, while defining the fitted amplitude factor as ‘SO_2_’ and fixing the value of N to 2.

### 3.3. HDF file-compatible functionalities

Since 2016, two files are systematically generated at completion of a given XAFS spectrum at SAMBA. The first one is an ASCII file, which essentially features, in specific data columns, the energy and absorption data of the XAFS spectrum, so that the latter can be readily imported into any XAFS data treatment software. The second file is an HDF file, which contains more thorough and detailed information relative to the collected XAFS spectrum. It notably features multiple contextual information, including the general configuration of the beamline acquisition system, scan parameters, setup of monochromator and its associated stabilizer device, and position values of all remotely readable equipment of the beamline (*e.g.* motors, thermocouples, clock,…) at the start and end of the XAFS spectrum. The HDF file also contains all raw data collected by the beamline multi-pixel fluorescence detector, *i.e.* a 36 pixel Ge detector (Canberra) or 13 pixel silicon drift detector (Mirion). This includes, for each pixel of the detector and each energy of the XAFS spectrum, the dead time, input and output count rates, and the multi-channel analyser (MCA) pattern.

**Figure 7:**
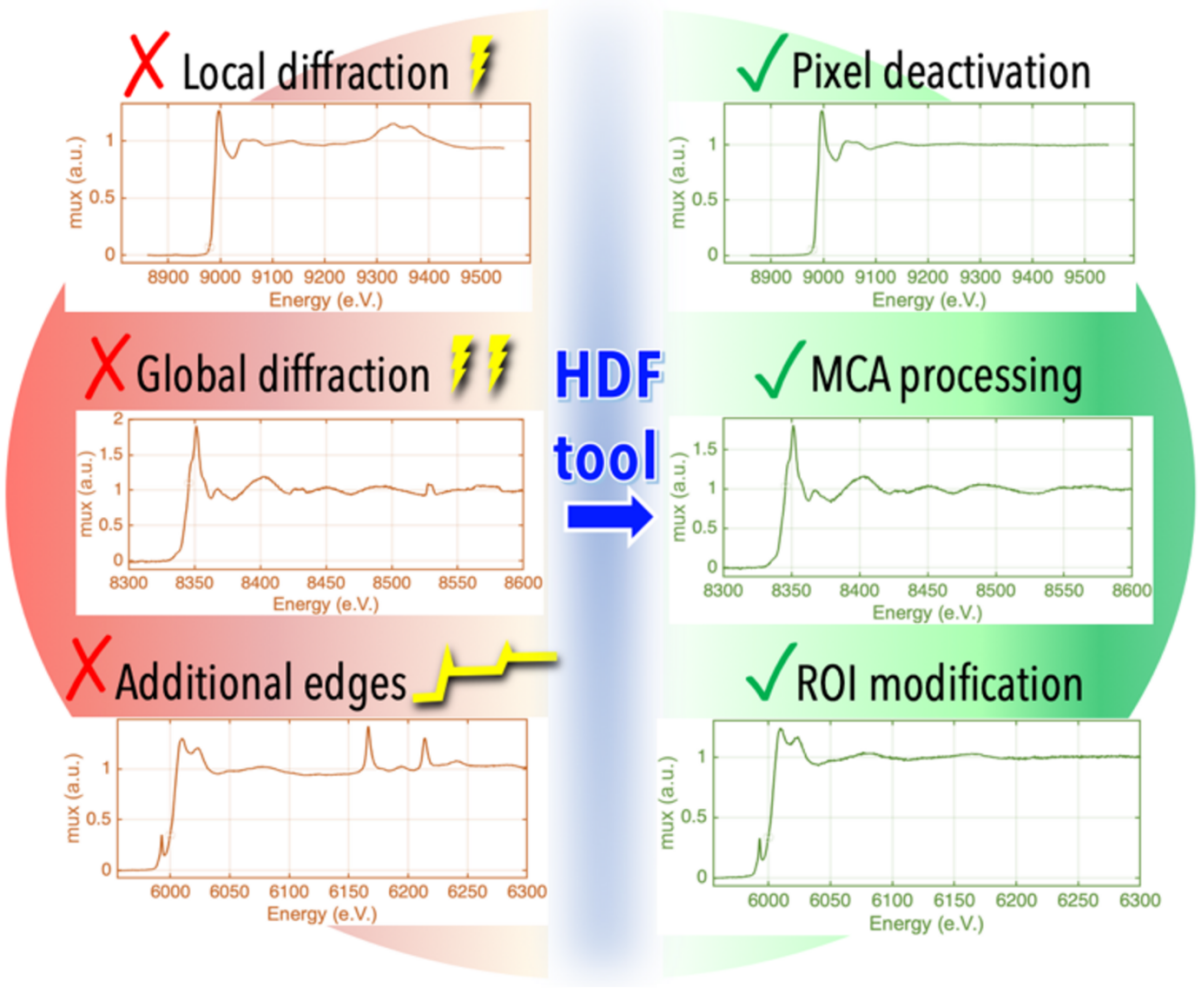
Examples of XAFS spectra affected by artefacts and acquired at the beamline (left) and new XAFS spectra (right) obtained post-beam-time using the tools available in *Fastosh* to exploit the raw fluorescence data saved in the HDF file.

*Fastosh* features functions that allow SAMBA users to easily access and exploit the data saved in the HDF file. This includes GUIs to display all contextual information (Fig. S9). For example, it is possible to show, in a 2D plot, the temperature of an oven over time for all scan iterations recorded during an *operando* experiment carried out at the beamline [Fig. S9(*b*)]. Additionally, some tools can be utilized post-beam-time to extract a new, artefact-free XAFS spectrum, using the MCA data saved in the HDF file. This may be necessary when the original spectrum, acquired at the beamline in fluorescence mode, is affected by artefacts and consequently inexploitable. For example, when not all pixels of the fluorescence detector are affected by diffraction phenomena, a new XAFS spectrum may be obtained without utilizing the data collected by the problematic pixels (Fig. 7 ▸ and Fig. S10). Alternatively, when diffraction phenomena affect all pixels of the detector, an artefact-free XAFS spectrum may be extracted by exploiting the part of the MCA below the region of interest (ROI) (Fig. 7 ▸ and Fig. S11). Indeed, diffraction from the sample crystalline phases produces several types of spectral artefacts; some of them arise from an extra fluorescence contribution from the diffracted beam. They may be attenuated or completely removed considering that lower energy fluorescence lines from the sample itself are non-EXAFS modulating, but are equally affected by diffraction. Lastly, when fluorescence emissions corresponding to elements other than the one studied contribute to the counts recorded in the ROI, the latter can be reduced, prior to extraction of a new XAFS spectrum from the MCA, to minimize the secondary fluorescence contributions (Fig. 7 ▸ and Fig. S12).

## 4. Conclusion

The main current features of *Fastosh*, including multiple functions that are unique to the program, were showcased. Given that the software is still under active development, it is possible to subscribe to an email list available on SAMBA’s website (https://www.synchrotron-soleil.fr/en/beamlines/samba) to be notified of any future version releases or important news related to *Fastosh*. While recent advanced data treatment methods, such as those relying on artificial intelligence, may be implemented in the program later on, a module dedicated to formal shell-by-shell fitting of the EXAFS, with unique functionalities that can optimize and facilitate such important data treatment approach, could be added to *Fastosh* in the near future. Finally, some of the benefits of saving metadata (*e.g.* contextual information and raw fluorescence data) relative to a single XAFS acquisition were demonstrated. Hopefully, this will help promote the use of such a data-saving approach and the adoption of a yet-to-be-defined universal HDF format by the international XAFS community.

## Supplementary Material

Additional figures: Figs. S1 to S12. DOI: 10.1107/S1600577525003923/rv5190sup1.pdf

## Data Availability

The data supporting the results reported in this article can be accessed upon reasonable request, except the Cu kinetics dataset, which does not belong to the authors as mentioned in the *Acknowledgements* section.
