# Supplementary material for: Fastosh: a software for the treatment of XAFS datasets of environmental relevance or acquired in operando conditions
Source: J Synchrotron Radiat. 2025 Jun 23;32(Pt 4):1085–94. doi: 10.1107/S1600577525003923 (PMC12236239; doi:10.1107/S1600577525003923)
Supplement: Supplementary file 1 [file s-32-01085-sup1.pdf]

JOURNAL OF SYNCHROTRON RADIATION

Volume 32 (2025)

Supporting information for article:

Fastosh: a software for the treatment of XAFS datasets of environmental relevance or acquired in *operando* conditions

Gautier Landrot and Emiliano Fonda

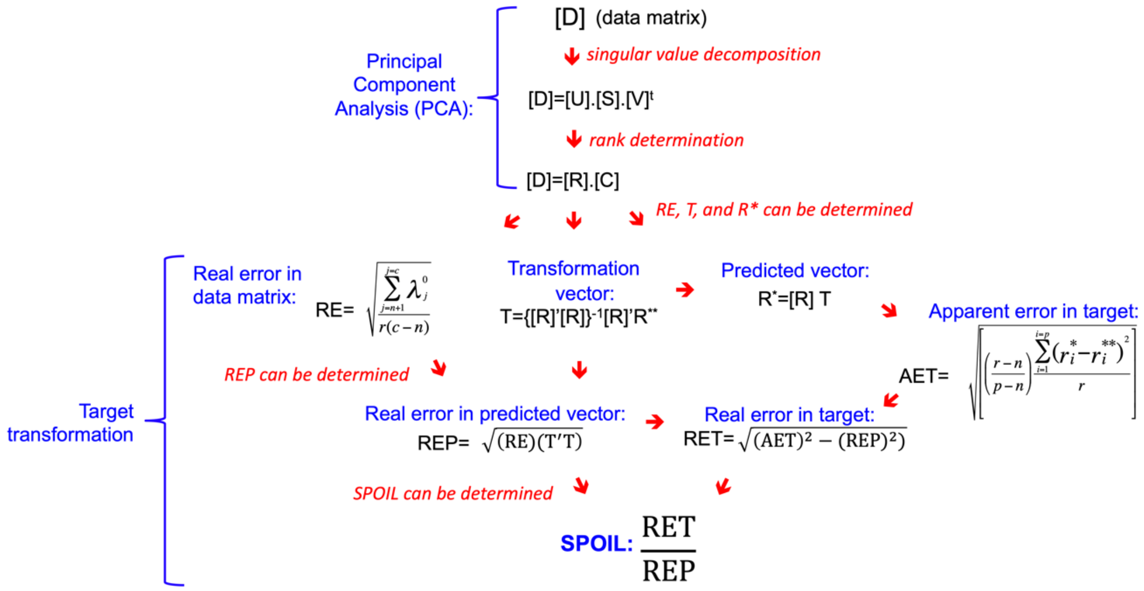

Figure S1 Schematic representation of the SPOIL calculation procedure via Principal Component Analysis and Target Transformation as introduced and detailed in Malinowski (1978).

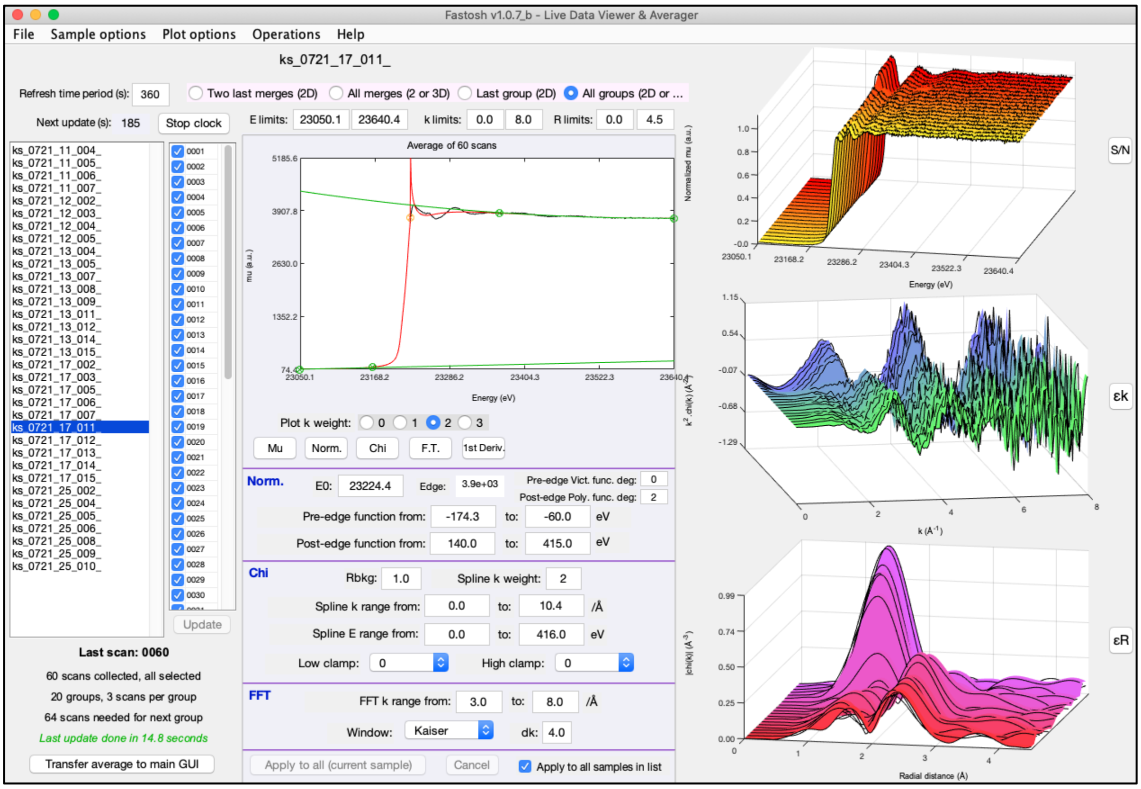

(a)

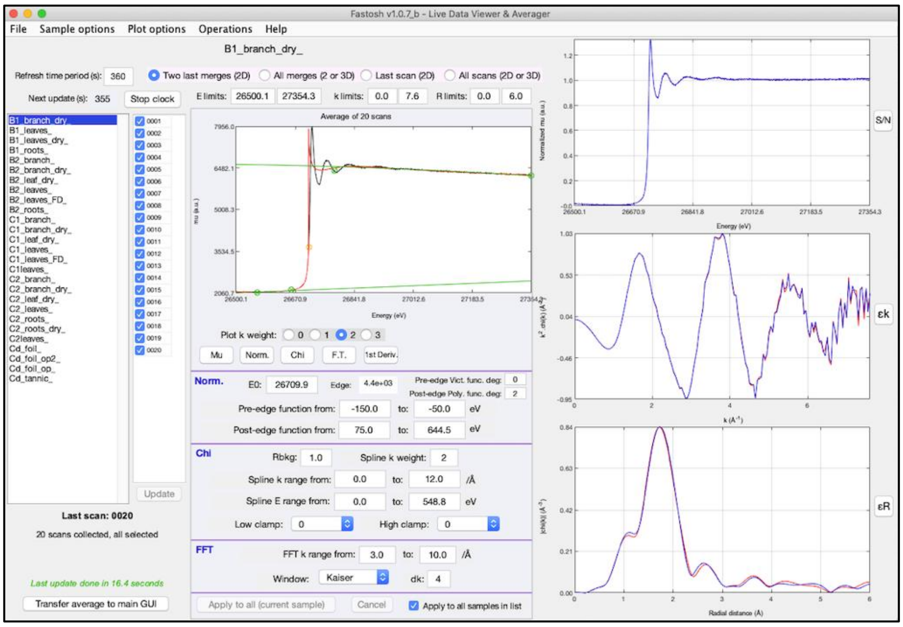

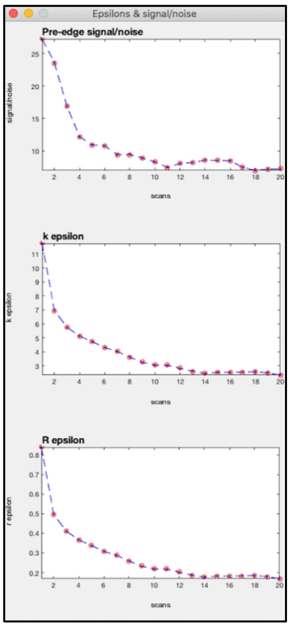

(b) (c)

Figure S2 Live Viewer & Merger GUI. It is refreshed automatically and periodically following a countdown displayed in the top left corner of the window. It can be employed for instance to follow in real-time the progress of a kinetics experiment (a), or visualize the data relative to a diluted sample (b). A secondary window shows the progressive improvement of the estimated random noise and signal-to-noise ratio for a set of spectra corresponding to a specific sample (c).

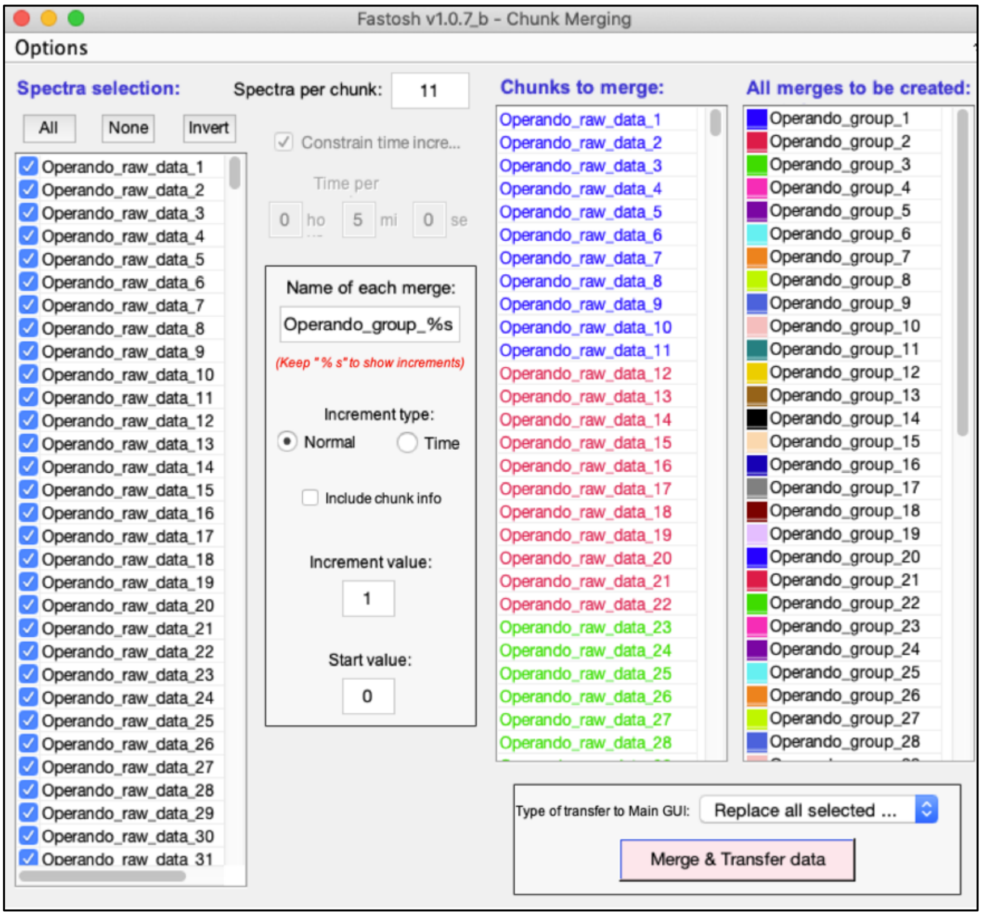

Figure S3 Chunk merging GUI: example where a dataset constituted of 616 XAFS spectra collected in *operando* conditions at a quick-XAFS beamline was reduced to 56 spectra, by merging every 11 spectra consecutively acquired at the beamline.

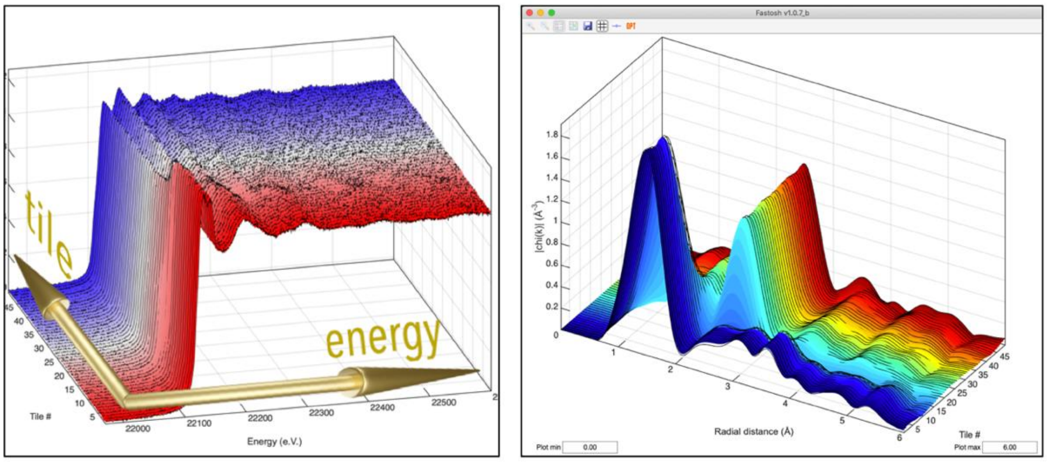

(a) (b)

Figure S4 Same dataset as the one shown in Figure 1, prior to applying a 2-D filtering in the energy and tile direction on the normalized spectra (a), and in Fourier Transform space after applying a 2-D filter (b)

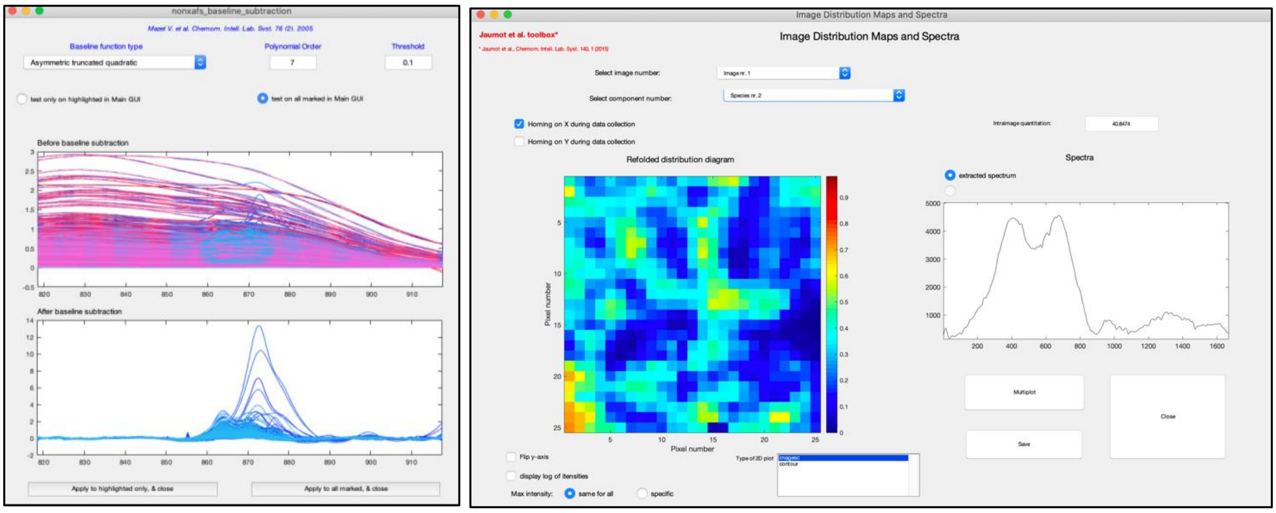

Figure S5 Processing in Fastosh of a RAMAN dataset acquired in 2D by a benchtop RAMAN spectrometer: GUI for baseline subtraction based on the method of Mazet *et al.* (2005) (a) and subsequent MCR-ALS treatment using a customized version of the toolbox of Jaumot *et al.* (2014).

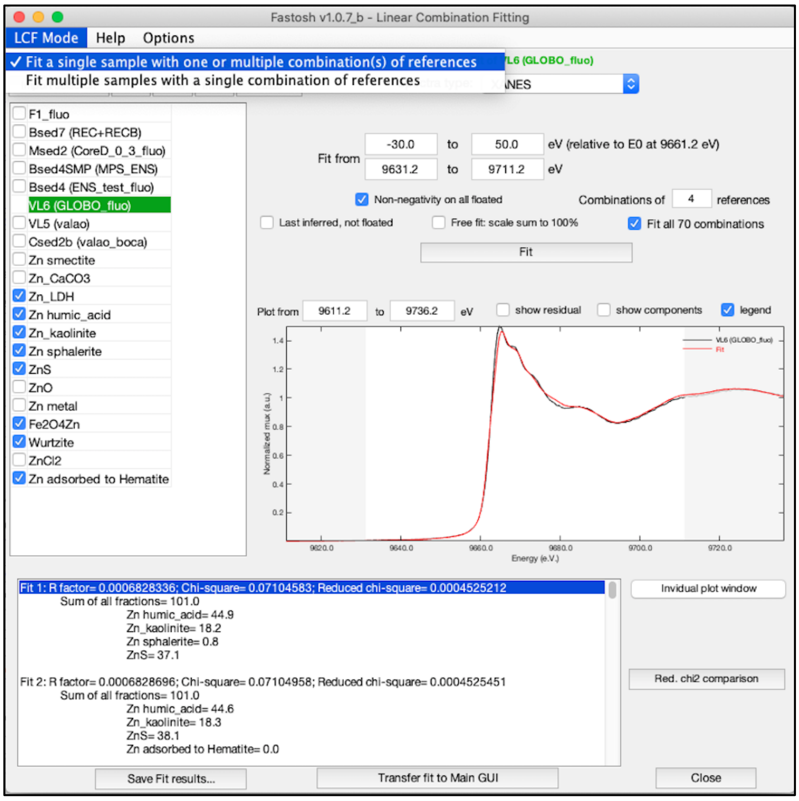

(a)

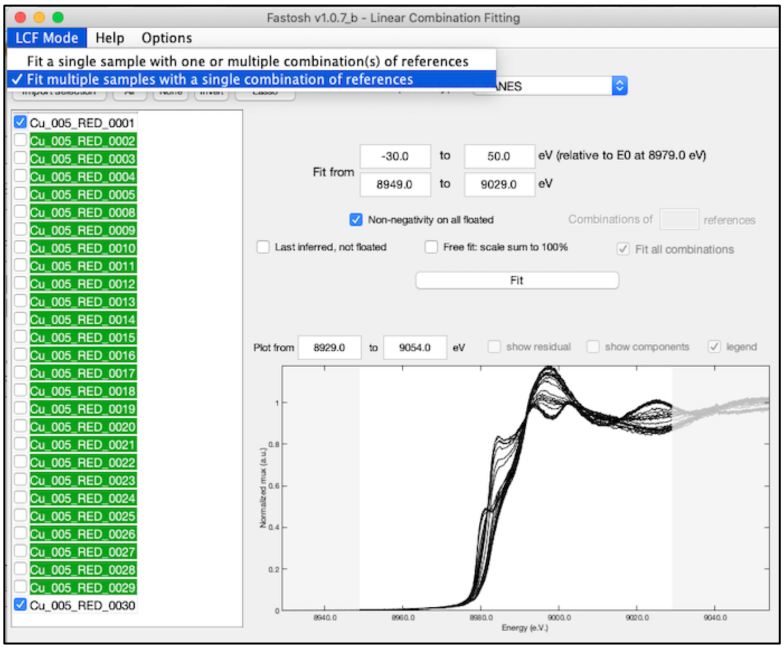

(b)

Figure S6 Two modes are available to perform Linear Combination Fitting (LCF): a single sample spectrum is fitted by LCF using multiple combinations of references (e.g. 8 references are selected from the list and 4 references per combination are defined, then 70 LCF operations are performed) (a), or multiple samples can be fitted by LCF using a single set of references (e.g. LCF is performed on all spectra of a kinetics dataset, from the 2nd to the 27th iteration, using as references the 1st & 28th (i.e. first & last) iteration of the dataset) (b).

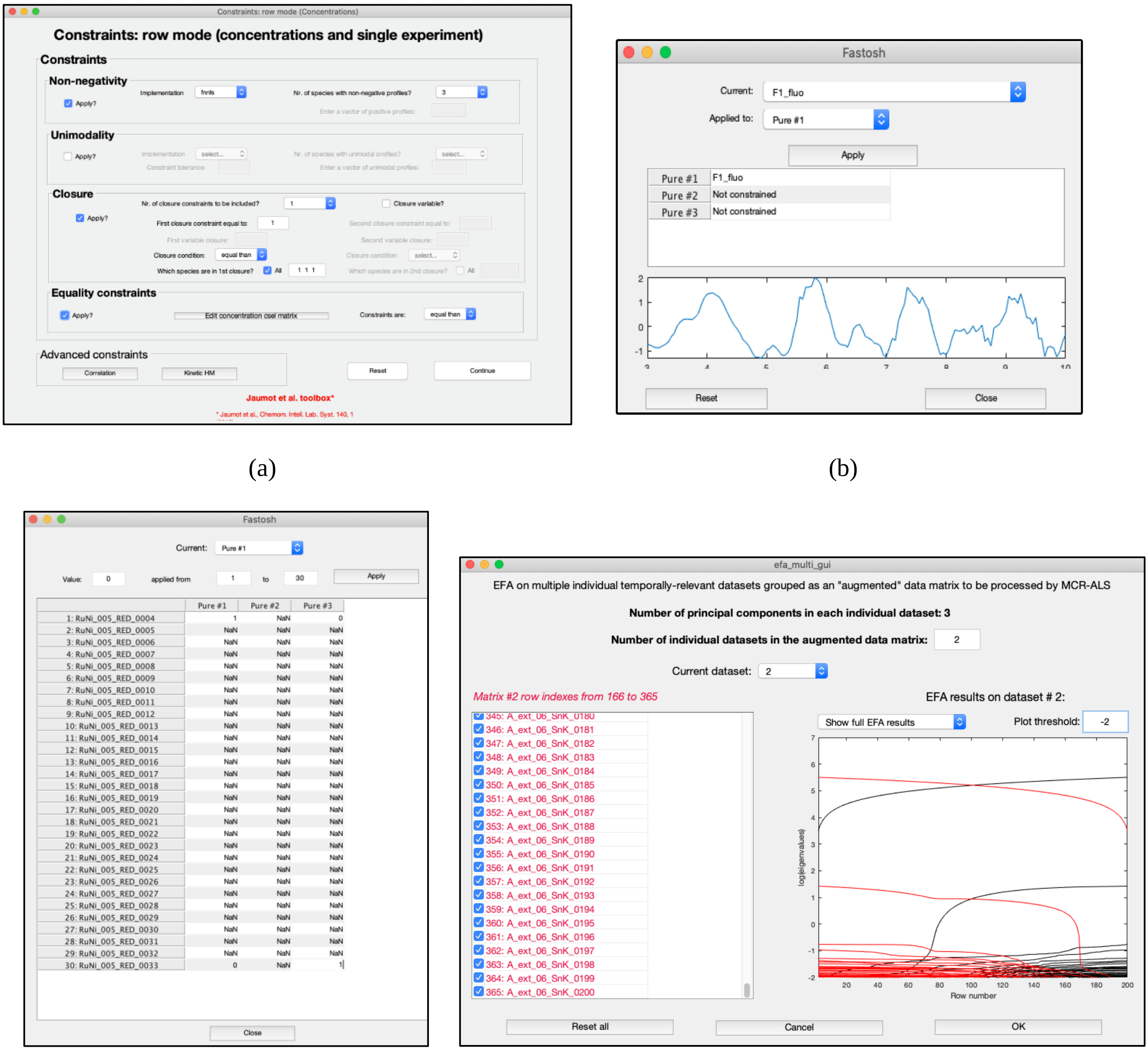

(a) (b)

(c) (d)

Figure S7 Custom features added to the MCR-ALS toolbox of Jaumot et al. (2014): the typical ALS constraints are automatically set to a mu dataset (i.e. the non negativity and closure constraints applied to the coefficient matrix + non negativity constraint applied to the spectral matrix) or EXAFS dataset (i.e. the non negativity and closure constraints applied to the coefficient matrix) (a); added GUI for interactively constraining the spectrum of each pure species (b) or the coefficient in a sample of each pure species (c); and added GUI to perform the Evolving Factor Analysis (EFA) on an “augmented” dataset (d)

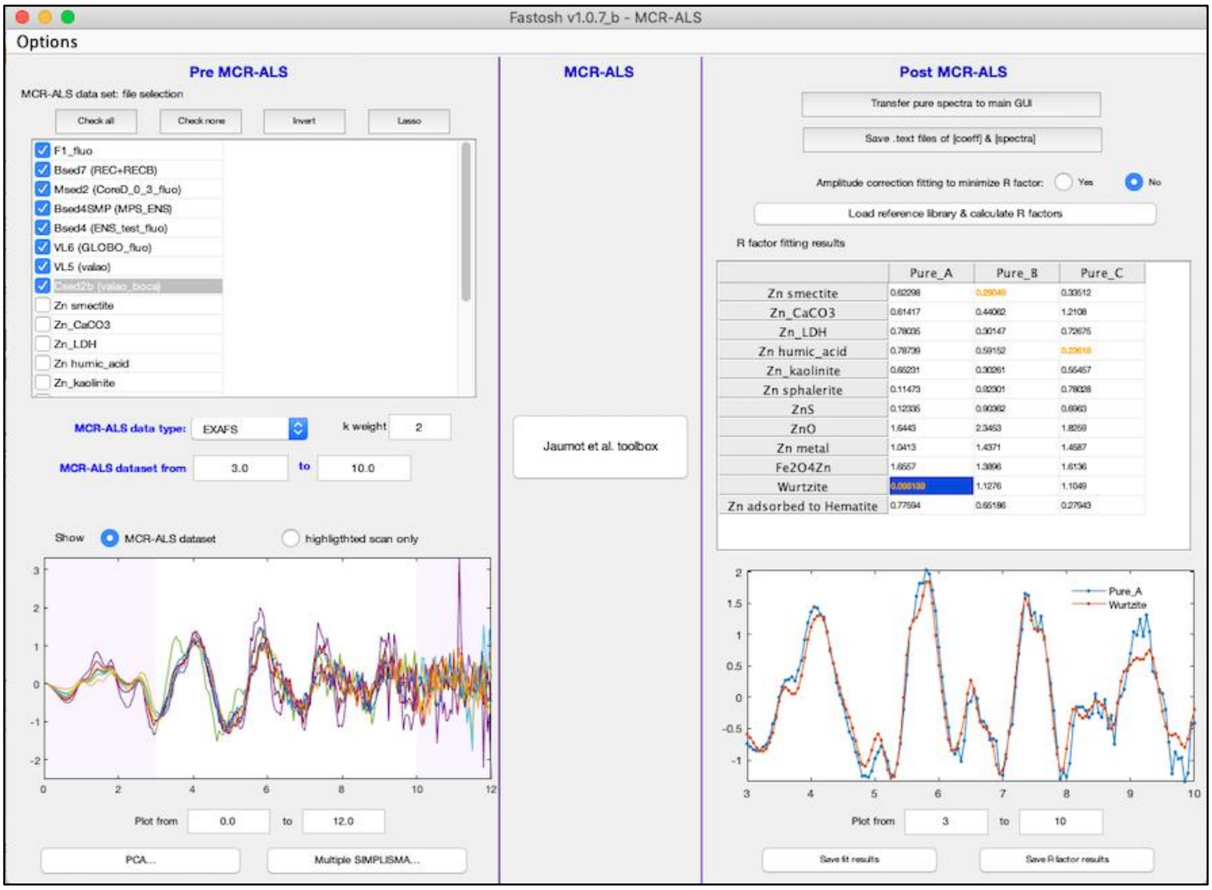

Figure S8 The MCR-ALS main window features, on its right side, a post-MCR-ALS functionnality that can help identify the nature of the MCR-ALS-extracted spectra, corresponding to the pure species of the mixture, by quantitatively comparing them, by mean of R factor calculation, to those belonging to a personal standard library. In the R factor result table, the reference that provided the lowest R factor value for a given pure species is highlighted with a yellow color. Data from Garnier et al. (2024).

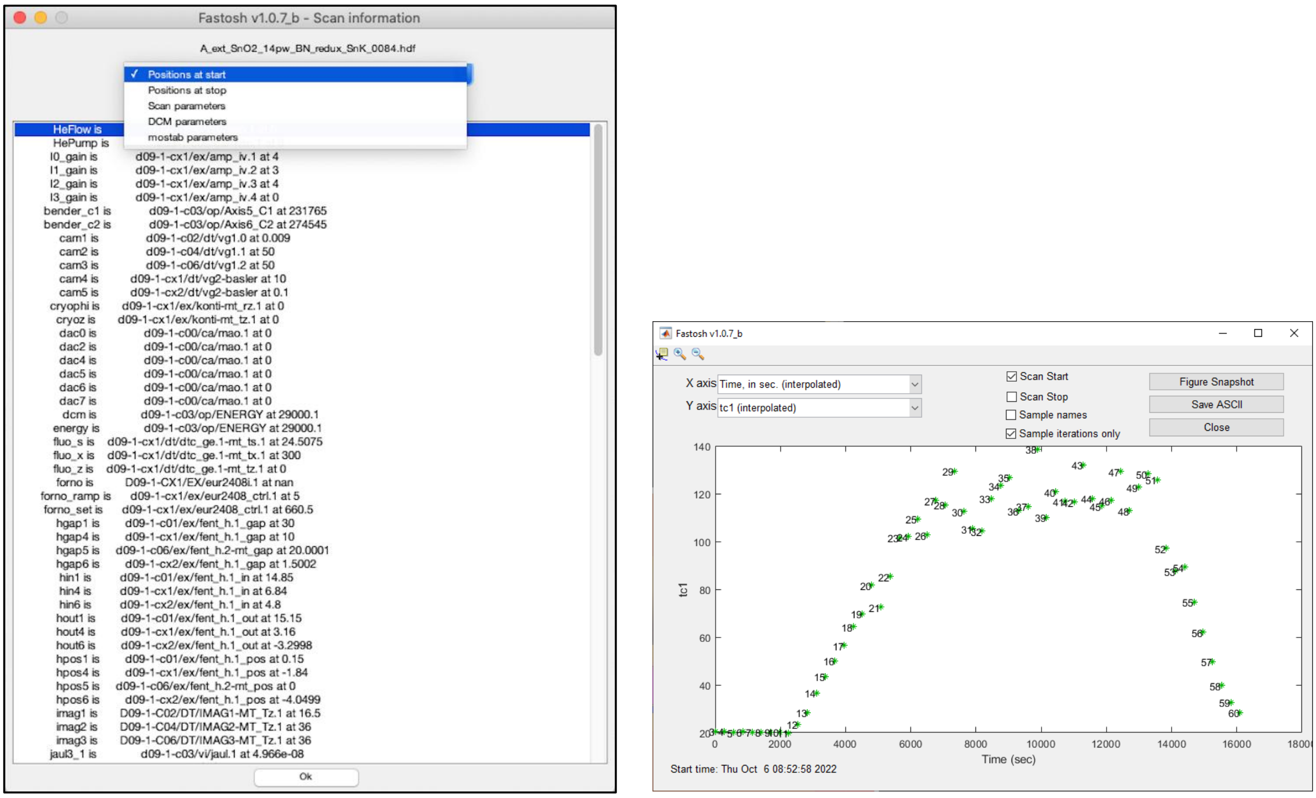

(a) (b)

Figure S9 Examples of GUIs available in Fastosh to display the contextual information saved in a SAMBA HDF file associated to a given XAFS spectrum acquisition: window to display the positions of all beamline readible equipments at start of the acquisition (in this window, other types of contextual information are accessible via the top menu, as shown) (a), and window to create a 2D plot relative to all spectra selected in the main GUI (here, the temperature of an oven is plotted over time, for all scan iterations recorded during an *operando* experiment carried out at the beamline)

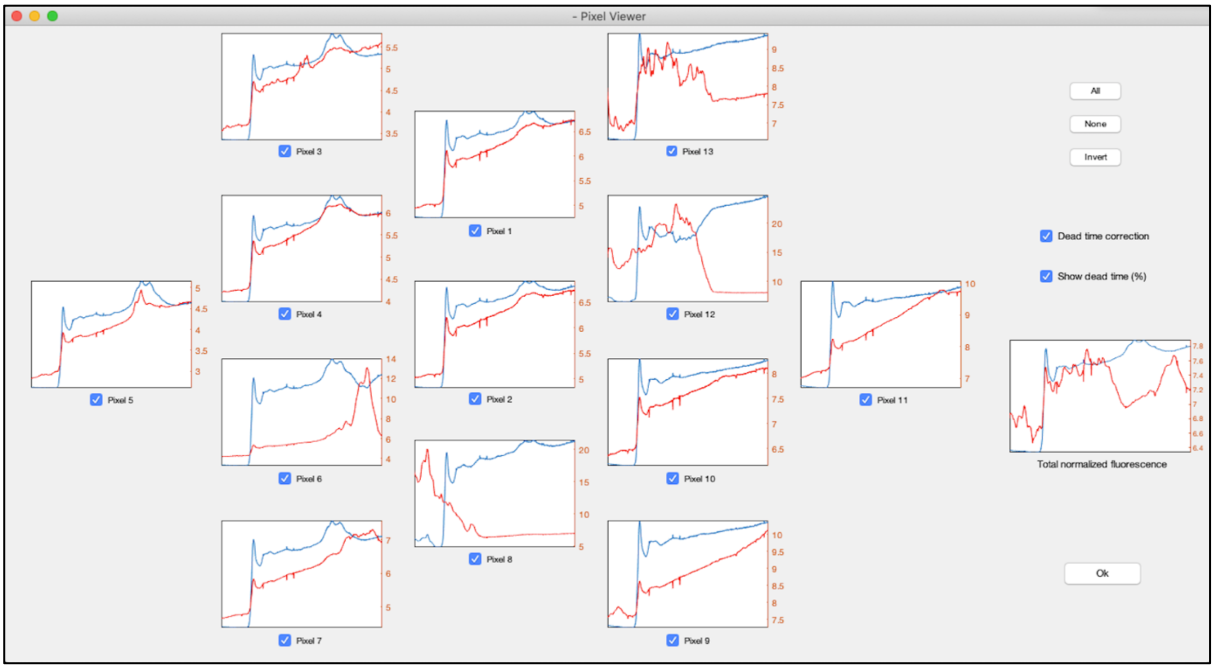

(a)

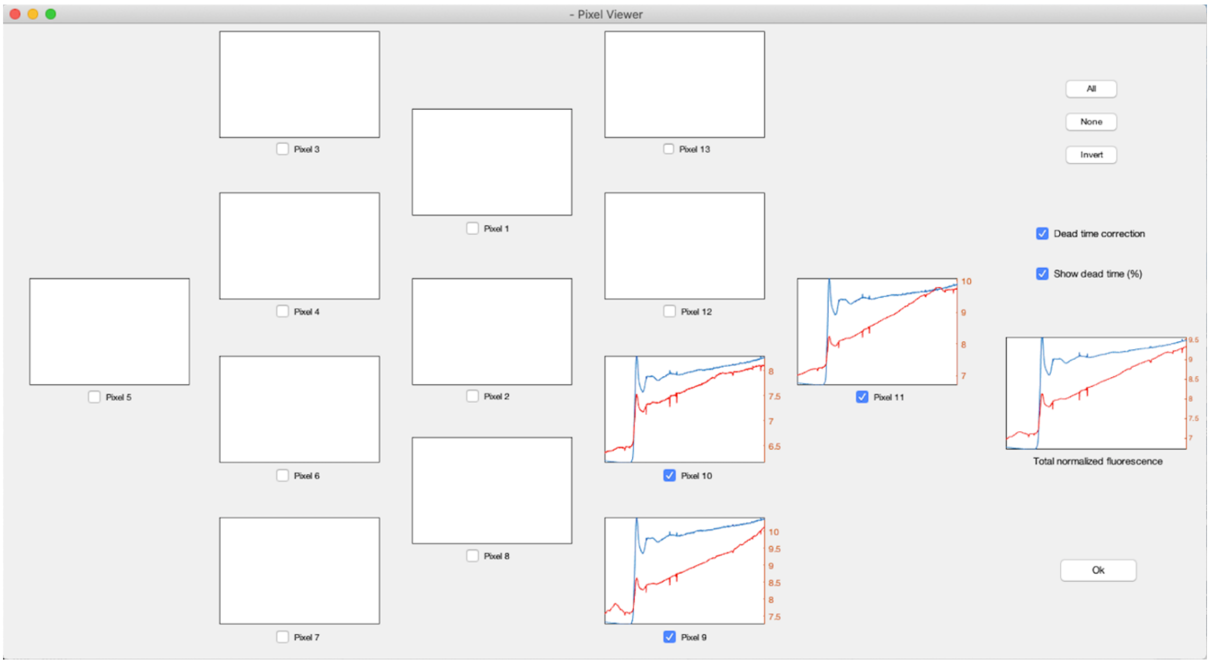

(b)

Figure S10 Example of a problematic XAFS spectrum acquired in fluorescence mode where diffraction phenomena significanly affected some of the pixels of the detector (a). A new, artefact-free XAFS spectrum could be obtained post-beamtime using the data saved in the HDF file after unselecting the problematic pixels in a dedicated Fastosh tool (b). In the above example, deglitching was subsequently performed on the new extracted spectrum (not shown).

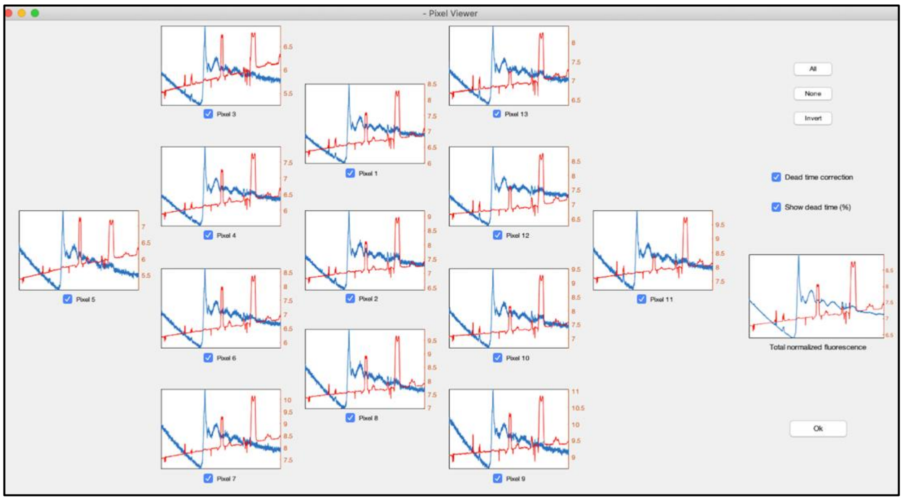

(a)

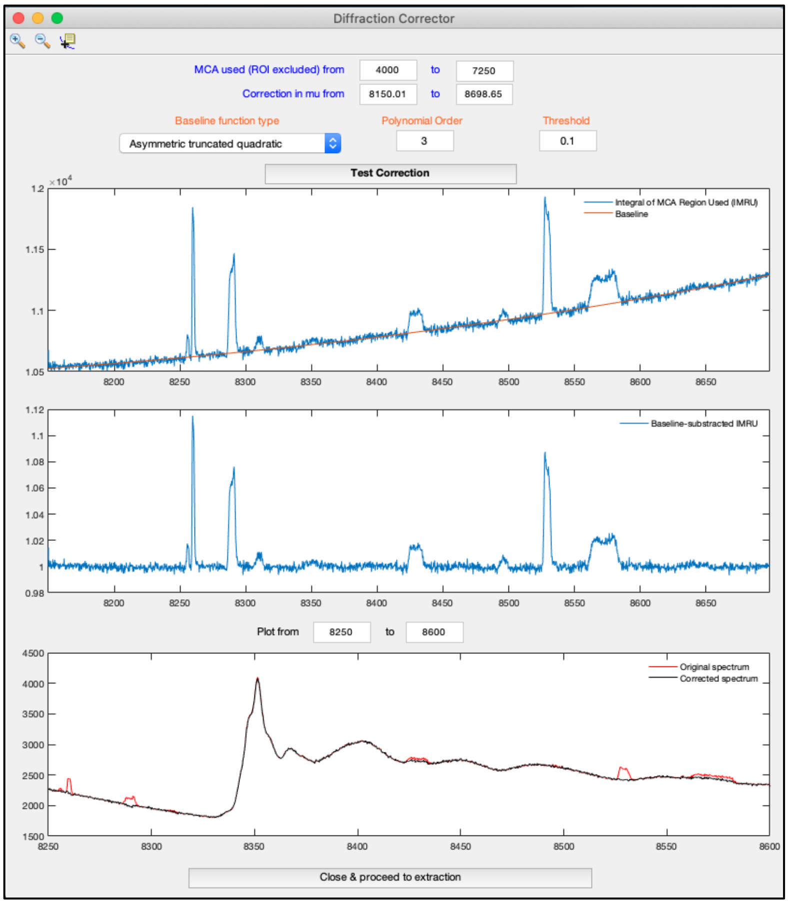

(b)

Figure S11 Example of a problematic XAFS spectrum acquired in fluorescence mode where diffraction phenomena affected all pixels of the detector (a). Therefore, the method described in Figure S9 could not be employed. A new XAFS spectrum with less artefacts could be obtained post-beamtime using the data saved in the HDF file, and after employing a Fastosh tool where the original XAFS spectrum was subtracted by an array corresponding to the integral of a MCA region (i.e. 4000-7250 eV) below the one corresponding to Region Of Interest (ROI) employed to acquired the original spectrum (7300-7620 eV), and at each energy of the XAFS spectrum (b).

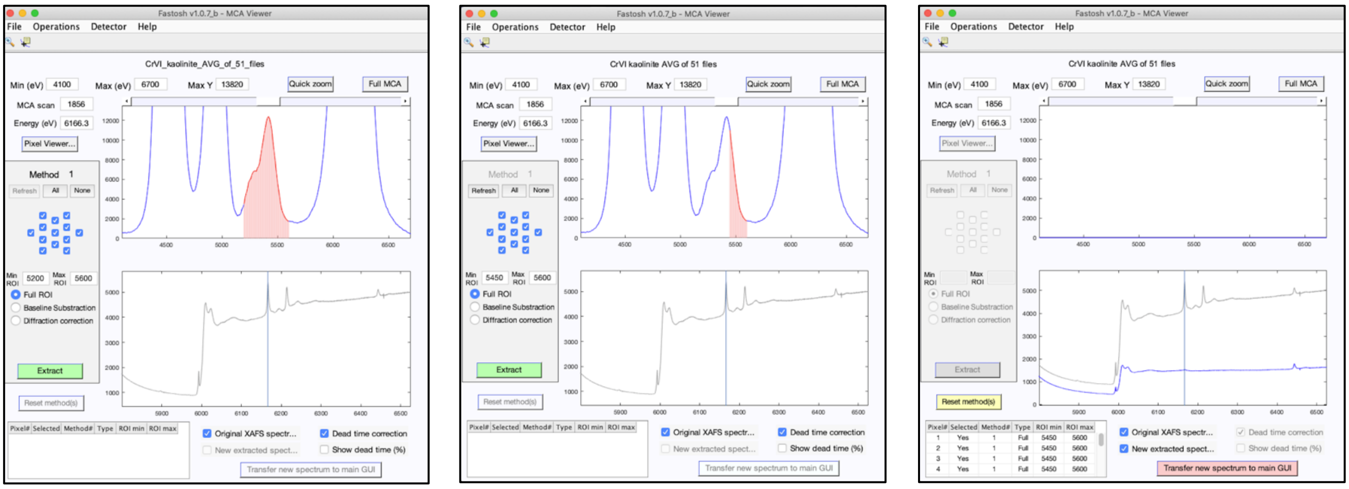

(a) (b) (c)

Figure S12 Example of a problematic XAFS spectrum acquired in fluorescence mode where a secondary emission line contributed to the counts measured in the Region Of Interest (ROI) employed to acquired the original spectrum (5200-5600 eV). After reducing the ROI to 5450-5600 eV using the data saved in the HDF file and a dedicated Fastosh tool (b), a new XAFS spectrum could be obtained (c). In the above example, truncating and deglitching was subsequently performed on the new extracted spectrum (not shown).

References

Garnier, J., Tonha, M., Araujo, D. F., Landrot, G., Cunha, B., Machado, W., Resongles, E., Freydier, R., Seyler, P. & Ratié, G. (2024). *Journal of Hazardous Materials* **480**, 135714.

Jaumot, J., De Juan, A. & Tauler, R. (2014). *Chemometrics and Intelligent Laboratory Systems* **140**.

Malinowski, E. (1978). *Analytica Chimica Acta* **103**, 339-354.

Mazet, V., Carteret, C., Brie, D., Idier, J. & Humbert, B. (2005). *Chemometrics and Intelligent Laboratory Systems* **76**, 121-133.
